# Purposes of internet use among Iranian university students: exploring its relationship with social networking site (SNS) addiction

**DOI:** 10.1186/s40359-022-00745-4

**Published:** 2022-03-26

**Authors:** Yasaman Hashemi, Fariba Zarani, Mahmood Heidari, Khatereh Borhani

**Affiliations:** 1 Department of Psychology, Shahid Beheshti University, Tehran, Iran; 2 Institute for Cognitive and Brain Sciences, Shahid Beheshti University, Tehran, Iran

**Keywords:** Internet use, Social networking, Addictive behaviors, Health education, Survey

## Abstract

**Background:**

Higher education students are heavy users of the internet for a wide variety of reasons, including Social Networking Sites (SNSs). This study investigated various purposes of internet use among undergraduate university students, and how different categories of such activities are related to SNS addiction.

**Methods:**

The sample set from 420 SNS users (280 females), a survey questionnaire was used to collect the information, including demographic information, purposes for internet usage (social, entertainment, academic, and economic purposes), and SNS addiction. Descriptive statistics, correlation, and a path diagram to estimate the regression coefficients were used to examine the data.

**Results:**

Findings revealed that the most common purposes for using the internet were online social networking and information seeking, followed by listening to music or watching movies, learning, relaxing, using email for educational needs, and reading socio-political news. In the comparison between categories of purposes for internet usage, the most prevalent group of purposes were academic and informative, recreational, social, and economic categories respectively. Moreover, the most significant influences belonged to the social group [positively], the academic and informative group (negatively), and the recreational group (positively) on SNS addiction respectively. The economic group of purposes did not have a significant influence on SNS addiction.

**Conclusion:**

This study has important implications for education and health providers, particularly in universities; we recommend that they try to improve students’ mental health and academic performance by providing opportunities for them to improve their IT literacy and skills.

**Supplementary Information:**

The online version contains supplementary material available at 10.1186/s40359-022-00745-4.

## Background

Internet has become an integral part of living in the contemporary societies and has globally altered many aspects of people’s lives. The number of internet users in Iran has increased considerably, from 3.8% in 2000 to more than 69.1% in 2018 [1]. The highest prevalence of internet use has been reported in many surveys to be among younger and more educated individuals [2]. Higher education students are heavy users of the internet for a wide variety of reasons. Using the internet with different goals could provide different opportunities and benefits for students [3].

Simultaneously, many studies have reported and discussed the negative impacts of pervasive internet use in students' lives [4, 5]. Researchers are becoming increasingly interested in maladaptive patterns of internet-related behaviors as a type of behavioral addiction [6]. Several studies have assessed the influence of different online activities on internet addiction as a whole concept [7, 8], as opposed to subtypes of internet addiction. Widyanto and Griffiths [9] emphasized on the decisive role of various online behaviors in the formation of addiction, and empirical results highlighted the importance of differentiating among specific types of internet application and their potential pathological usage [10]. Furthermore, recent literature has revealed the need to gain a better understanding of students’ online behaviors, based on diverse categories of internet usage [11].

SNSs are virtual communities for sharing common interests and SNS addiction is a specific subtype of Internet addiction [12]. With the growth of the internet over the years, the ways of communication and socialization have changed, especially via SNSs. Since different forms of online behaviors (even those like information-seeking) are often linked to social networking, Park et al. [13] considered social networking as one of the most significant online activities among the youths. Moreover, they showed a positive relationship between online activities, the diversity and size of social networks, and the way young people engaged in social networking. Given that the role of various categories of internet usage in SNS addiction is still unclear, this study aims to investigate purposes of internet use among Iranian university students and to explore how different categories of such activities are related to SNS addiction.

## Purposes of internet use among university students

Tracking the evolution and increasing penetration of the internet, researchers reported changes in students’ internet use patterns. Jones et al. [14] discussed these changes over time and reported an increase in internet use with entertainment goals, and a decrease in internet use with homework goals from 2002 to 2005. Social communication, entertainment, coursework, and professional communication were the most common reasons for internet use. Another study showed the most prevalent purpose for Turkish students’ online activities was entertainment including chatting and social networking, downloading movies or music, playing games, gambling or shopping, followed by searching for homework, getting information for academic purposes, and using e-mail services [15]. A large number of studies illustrated internet as a functional tool that has extremely changed students’ ways of interactions with others and their access to information and academic resources. Recent literature has recorded engagement in social activities, sharing common interests, gaining new experiences, dispensing with boredom, enjoying entertainment, and responding to other needs as purposes of internet use in students [8].

Besides, internet has completely changed the learning context of university students [16], as many students use novel technologies for education-related purposes. Regarding the essential role of information technology (IT) in reaching educational goals, several studies have addressed the positive impacts of computer and internet skills and digital literacy on students’ academic achievements [17, 18]. On the other hand, some studies have concerned about pathological or addictive internet-related behaviors (including SNS addiction) and their negative impacts on academic performance [19]. Although some researchers showed an increasing interest in the academic use of social media in students [20], to the best of our knowledge, there are no studies to investigate the relationship between internet use for academic purposes and SNS addiction.

## Purposes of internet use and SNS addiction

Researchers have tried to identify the typology of internet use and to offer its differentiated types [3, 21]. However, there exists an interrelationship between different classifications of internet use. Lee and Wu [22] for instance, categorized online reading into information-seeking and social-entertainment activities. Another instance is the social nature of many online games that has been described as an extra attractive aspect of them [23]. As a result, examining the relationship between some of these overlapping factors seems essential.

Online social networking is a critical part of youths’ social life, with an increasing influence and prevalence in students [24]. Because they are accessible and easy to use, SNSs have been highly embraced for a variety of social activities. Researchers confirmed an increased involvement in civic and political issues through SNSs [25].

Social needs are known to be the main reasons for SNS use. At the same time, SNSs greatly affect other personal needs. Park et al. [26] used factor analysis to classify the need for participating in Facebook groups into socializing, entertainment, self-status, and information-seeking. Entertainment was proved to be one of the main motives of SNS usage in several studies [27]. Dew and Tulane [28] categorized SNSs as a subtype of entertainment media. Kim et al. [29] found cultural differences in motivations behind SNS use; although students from both studied countries had some shared motivations such as seeking friends, social support, entertainment, information, and convenience, the Korean students put more emphasis on social desires, while for American students seeking entertainment was the most powerful incentive. According to Stockdale and Coyne [30], SNS use to the aim of alleviating boredom increases over the first years of adulthood and is also associated with SNS problematic use.

In addition to that, there is a large body of literature on ways through which SNSs leads to a more vibrant academic environment, such as the ease in sharing and collecting information, collaborating with classmates and professors, making study groups [31]. Alternatively, some researchers have considered academic activities as the most overlooked group of life activities in excessive SNSs use [32]. Lau [33] found SNS usage for non-academic purposes only (particularly gaming) to be a negative predictor of academic performance in university students. Recently, a negative relationship between the overall SNS use and academic performance was reported in Iranian medical students [34].

Various types of economical use of SNSs is another topic that attracts the attention of researchers, who believe in redefining business behaviors through social media and thus, discuss the advantages and challenges of SNSs in commercial-related communications [35, 36]. The significant role of entertainment, interactions, and trendiness created by social media was recently revealed at the luxury brand marketing [37]. At the same time, there is a scarcity of investigations on economical-related use of SNSs in students, even though shopping is one of the major goals of internet use among this group [15].

From there, the current study has two goals:

To compare the frequency of various purposes for internet use among university students.

To explore categories of purposes for internet use that contribute to SNS addiction scores among university students.

## Methods

### Participants and procedure

A total of 464 undergraduate students from Shahid Beheshti University in Tehran, Iran voluntarily participated in the study (in April and June 2019). They all had internet access and have been social media (e.g., WhatsApp, Telegram, Instagram, Facebook, and YouTube) users and were selected using a stratified sampling method (total number of students in different departments was taken into consideration). A pencil and paper survey in Persian was administered in classrooms by the researchers of this study. After excluding those with missing responses, 420 questionnaires included in the analysis (response rate: 90.5%). Among the respondents, 280 were females, and the age range was of 18 to 25 years (*M* = 20.09, *SD* = 1.63 years). Additional demographic information is listed in Table 1.

**Table 1:** Demographic information of study participants (n = 420)

Demographic characteristic	*n*	%
*Marital status*		
Single	408	97
Married	9	2
Other	3	.7
*Part-time job*		
Yes	88	21
No	332	79

Participants were fully informed of the purpose of the present study. Written consent forms were obtained from them. The study was approved by the ethics committee of the Shahid Beheshti University.

## Measures

The survey questionnaire comprised 37 questions in total. More specifically, the questionnaire examined the following:

### Demographic information

Questions 1 to 4 asked about demographic information, including age, sex (Female/Male), marital status (Single/Married/Other), part-time job (Yes/No).

### Purposes of internet use measurement

To assess purposes of internet use, participants were given a list of 13 questions and were asked to rate different reasons for their internet usage (social, entertainment, academic, and economic purposes). These variables were adopted from previous works [15, 21] and the choice of every item was based on the preliminary investigation on the most prevalent purposes known to 25 university students. Questions were on a Likert scale ranging from 0 = “*no use at all*” to 7 = “*Extreme use*”. Internal consistency was α = 0.7 for the whole scale. Table 2 shows the measures of the variables used in this section of the study.

**Table 2:** Purposes for internet usage measurement (n = 420)

Variable	Item	How often do you use the Internet for …?	%				*M*	*SD*
			0 or 1	2 or 3	4 or 5	6 or 7		
Social purposes	1	Meeting new people	59.3	21.2	13.6	6	1.66	.92
	2	Following social and political news	41.4	25.2	21.0	12.4	2.04	1.06
	3	Using SNSs	2.4	12.4	37.4	47.9	3.31	.78
Recreational purposes	4	Online gaming	66.9	15.2	10.0	7.9	1.59	.96
	5	Online gambling	94.5	2.4	1.7	1.4	1.10	.45
	6	Relaxing	12.4	27.6	36.2	23.8	2.71	.96
	7	Listening to music or watching movies	5.7	19.8	30.2	44.3	3.13	.92
Economic purposes	8	Shopping	43.3	33.6	17.4	5.7	1.85	.90
	9	Making money	86.9	7.1	3.1	2.9	1.22	.64
	10	Using online transportation services	49.0	18.1	17.6	15.2	1.99	1.13
Academic & informative purposes	11	Learning	7.4	24.3	41.2	27.1	2.88	.89
	12	Information searching	4.3	18.1	36.9	40.7	3.14	.86
	13	Using email for educational needs	36.0	28.1	22.4	13.6	2.13	1.05

(Scale ranging from 0 = “*no use at all*” to 7 = “*Extreme use*”)

### Internet addiction test-social networking sites version (IAT–SNS)

The tendency of pathological use of SNS as a specific form of internet addiction was assessed with the Internet Addiction Test (IAT; 38], modified for Social Networking Sites (IAT-SNS). We used Persian version of IAT [39], and modified it for SNS (by a wording change that replaced “Internet” with “Social Networking Sites” in each item). The IAT-SNS is a 20-item inventory. Respondents were asked to report how often they experienced obsession, compulsion, or problems related to the use of SNSs, through a 6-point Likert scale (0 = “does not apply” to 5 = “always”). The score range is 0 to 100, and with higher scores representing a greater level of pathological use of SNS. The cut-off points of 0–49 for normal users and 50 or above for probable problematic users are proposed for IAT-SNS [40]. Also, good internal consistency has reported for this scale (α = 0.92; 40]. The Persian version of IAT-SNS has shown good psychometric properties as well [41]. Cronbach’s Alpha for this study was 0.91.

### Data analyses

Using SPSS 26.0, descriptive statistics were performed to compare various purposes for internet use among university students.

Subsequently, correlation and regression analysis were used to explore the relationship between purposes for internet usage (independent variables) and SNS addiction (the dependent variable). Using Amos 26.0, Structural Equation Modeling (SEM) was performed, and the path diagram revealed the standardized estimates of the regression coefficient. Four categories of purposes for internet use (i.e., academic, recreational, social, and economic) were taken as exogenous variables. Also, sex as a potential moderator was entered into the model and was taken as an exogenous variable. SNS addiction was taken as an endogenous variable.

## Results

SNS addiction scores ranged from 2 to 82 (M = 32.41, SD = 15.18). The mean score was 32.10 for male students (SD = 15.29) and 32.56 for female students (SD = 15.15). Among participants, 355 (84.5%) were normal users of SNSs (scores 0–49), and 65 (15.5%) were problematic users (scores above 50).

### The frequency of purposes for internet use among university students

Based on data presented in Table 2, the highest rates of purposes for internet use belonged to social networking and information searching, followed by listening to music or watching movies, learning, relaxing, using email for educational needs, and reading socio-political news. The rating was lower for purposes of using online transportation services, online shopping, meeting new people, and gaming. Online money-making and gambling were the lowest rated purposes.

**Fig. 1:**
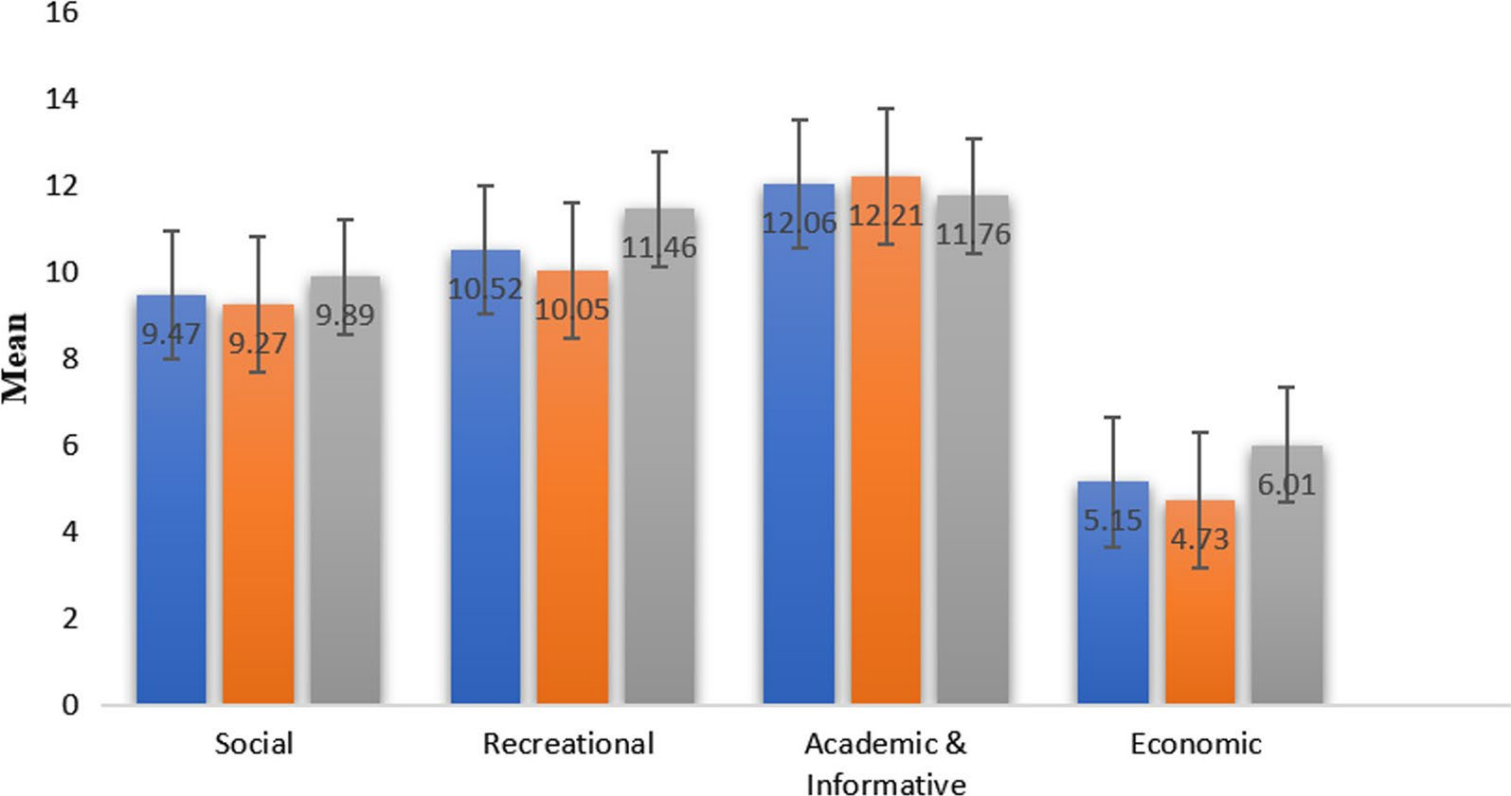
Distribution of students’ purposes for using internet. Error bars are denoting to SD

Figure 1 shows that the most prevalent group of purposes for internet usage among university students were academic and informative, recreational, and social categories respectively. The economic group was the weakest incentive for internet use among students. Yet, male students used the internet for recreational, economic, and social purposes more than female students.

### Relationship between purposes of internet use and SNS addiction

According to the findings presented in Table 3, there were significant and mainly positive correlations between various purposes of internet use. There was a positive and significant correlation between SNS addiction and the following purposes for internet usage: SNS use, meeting new people, and relaxing. Table 3 also shows a significant negative correlation between SNS addiction and internet use with the purpose of using email for educational needs.

**Table 3:** Bivariate correlations between questionnaire items and SNS addiction (n = 420)

SNS addiction	1	2	3	4	5	6	7	8	9	10	11	12	13
	0.20**	0.03	0.38**	0.09	0.02	0.26**	0.07	0.07	− 0.06	0.04	0.09	− 0.06	− 0.11*
1. Meet new people	–	0.20**	0.11*	0.03	0.20**	0.19**	0.11*	0.13**	0.23**	0.13**	0.06	− 0.01	− 0.02
2. Sociopolitical news		–	0.08	0.12*	0.14**	0.04	0.09	0.15**	0.13**	0.09	0.29**	0.29**	0.09
3. SNS use			–	0.10*	0.01	0.26**	0.14**	0.12**	− 0.20	0.12*	0.07	0.10*	0.00
4. Gaming				–	0.20**	0.12*	0.23**	0.18**	0.10*	0.15**	0.04	0.09	0.09
5. Gambling					–	− 0.03	0.17**	0.04	0.15**	0.19**	0.03	0.07	0.11*
6. Relaxing						–	0.23**	0.13**	0.03	0.17**	0.16**	0.20**	0.02
7. Movie & music							–	0.24**	0.04	0.23**	0.18**	0.25**	0.29**
8. Shopping								–	0.01	0.24**	0.60**	0.29**	0.34**
9. Making money									–	0.18**	0.10*	0.01	0.11*
10. Online transportation										–	0.13**	0.24**	0.25**
11. Learning											–	0.60**	0.34**
12. Searching												–	0.34**
13. Email for educational needs													–

*p < .05. **p < .01

**Fig. 2:**
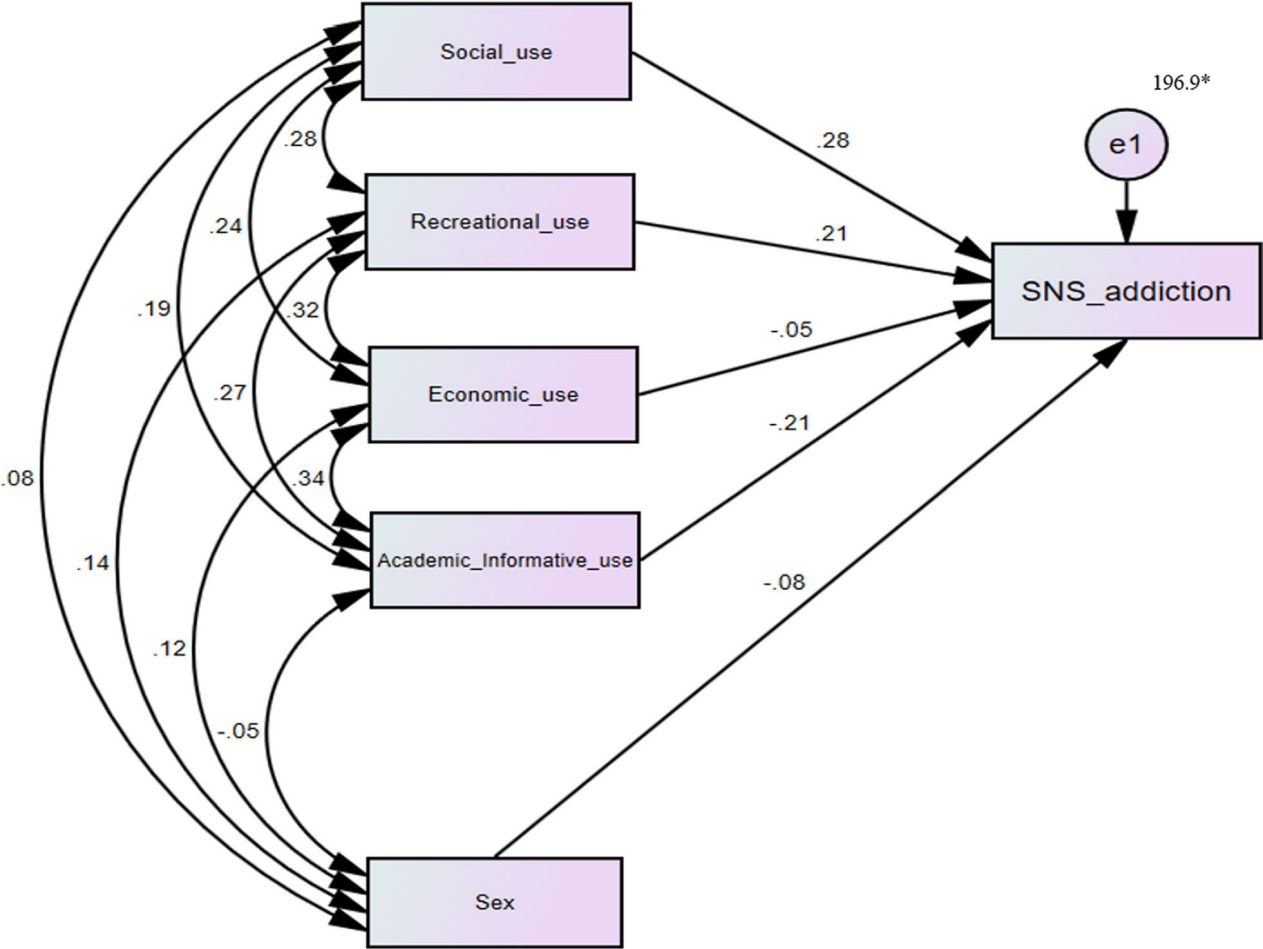
Modeling and interconnectedness estimation of purposes for internet use and SNS addiction. *Unstandardized estimate

The path diagram (Fig. 2) and Table 4 show the regression weights (with their significance levels) of social (0.272), academic and informative (− 0.206), recreational (0.190), and economic (− 0.034) purposes of internet use on SNS addiction. Among them, economic purposes category was not a significant predictor of SNS addiction. Also, sex did not have a significant effect on this model (see Table 4).

**Table 4:** Regression weights (n = 420)

	Purposes for internet use by category	Standardized estimate	Unstandardized estimate	S.E	C.R	*p*
	← Social use	0.275	1.098	0.191	5.754	***
SNS addiction	←Recreational use	0.209	0.733	0.175	4.194	***
	←Economic use	− 0.050	− 0.186	0.187	− 0.999	0.318
	←Academic & Informative use	− 0.208	− 0.714	0.169	− 4.221	***
	←Sex	− 0.075	− 2.417	1.478	− 1.635	0.102

***p < .001

## Discussion

### Internet use among university students

In this study, various purposes for internet use among university students were investigated. Findings revealed that the most common reasons for using the internet were online social networking and information seeking, followed by listening to music or watching movies, learning, relaxing, using email for educational needs, and reading socio-political news. On the other hand, using online transportation services, online shopping, meeting new people, and gaming were weaker incentives for internet use, and making money and gambling online were the least prevalent reasons for the internet use among university students. Moreover, in the comparison between categories of purposes for internet usage, the highest rate belonged to the academic and informative group. The recreational and social groups of purposes were second and third, and economic purposes were in the last place.

Our results correspond with those of some other studies that showed searching for scientific material [18] and academic issues [42] are students’ main goals for using the internet and corroborate studies that have addressed the importance of internet use skills and digital literacy on students’ academic achievements [17]. Likewise, Deniz and Geyik [15] found online chatting and membership in social networks as the most common and seeking friends as the least common purposes of internet use, and Khamis et al. [11] reported higher rates for entertainment and social media use purposes than for online shopping. They also found information-seeking to be one of the most common goals for internet use.

However, there are some discrepancies as well. Both above mentioned studies reported that entertainment purposes were preferred by university students to learning purposes. It should be noted that the finding of the study regarding the category of recreational usage not being much prevalent, might be due to the fact that some items in this category are considered illegal in Iran (e.g., gambling). In addition to cultural differences, this discrepancy may be due to the differences in the methods for assessing and categorizing internet use purposes. Khamis et al. [11] analyzed some secondary data from the university resources, as well as the frequency of visited websites categorized by the university, and Deniz and Geyik [15] assessed various internet use purposes through dichotomous questions. Besides, considering the rapid progress of digital technology, several researchers emphasized the importance of ongoing efforts for understanding the changes in internet use patterns over time [43]. Therefore, there still is a need for further investigation regarding categories of using the internet and its implications in different samples of students.

### Relationship between purposes for internet use and SNS addiction

Another contribution of this study lies in investigating how various purposes for internet use relate to SNS addiction. Among social purposes, only the purpose of following socio-political news did not have a significant correlation with SNS addiction. While SNSs are known as an increasingly attractive space for involvement in civic and political issues [25], it seems that this increasing involvement is not significantly correlated with pathological uses of SNSs.

In terms of categories of purposes for using the internet, the most significant influence belonged to the social group with a positive influence, followed by the academic and informative group with a significantly negative influence. After that was the recreational group with a significantly positive influence on SNS addiction. The economic group of purposes did not have a significant influence on SNS addiction. Similar to our findings, Aljuboori et al. [20] showed an increasing interest in the academic use of social media in students. They recommended focusing on academic aspects of students’ SNS use, while considering the potential negative impacts of issues such as the addictive use of SNSs on students’ health. Based on our results, academic and informative purposes of internet usage decrease the addictive use of SNSs. By considering the rapidly increasing impact of the internet on academic achievement, some researchers have concerned about the digital divide and the need to increase internet use-related literacy and digital skills [17]. Our results highlight this essential need in university students as well. Ergun-Basak and Aydin [8] also found students' using the internet for social media and games was more associated with problematic internet use, compared with using the internet with academic purposes.

As the results show, the social purposes group for using the internet had the most positive influence on SNS addiction. Previous studies declared social-related variables as the strongest reasons for SNS use [44]. Based on our findings, the social group of purposes for using the internet has a stronger effect on SNS addiction in Iranian university students than the recreational purposes group. These findings are similar to those of several other researches that show entertainment is the second strongest incentive for SNS use, after social reasons [45]. In this respect, Kim et al. [29] explored the role of cultural differences in determining whether social motives or recreational ones were the strongest incentives for student’s SNS use. To our knowledge, however, there is no previous study to explore intentions of SNS use in students in Iran.

The group of economic purposes of internet use did not have a significant influence on SNS addiction. This finding might be due to the nature of the sample group; before achieving a professional state, the economic purposes of SNS use could be less attractive for undergraduate students. Likewise, another recent study reported a low preference for using social media for business purposes in university students as well [20]. Further investigation is needed to better understand the commercial SNS use and its negative impacts on students.

This study had a number of limitations. First, the proposed model only examined the impact of 13 purposes for using the internet (categorized into four groups). Future studies could investigate more and different categories (e.g., sexual purposes of using the internet) in relation to SNS addiction. Considering some cultural aspects, we preferred to limit ourselves to more reliable purposes only. Second, due to the cross-sectional nature of the study, the causal relationships between variables may not be warranted. Longitudinal designs are needed to provide valid causal effects. Third, the population of this study was limited to university students, which are heavy users of the internet for a wide variety of reasons and at the risk of SNS addiction [24]. Therefore, generalizing results to other populations may not be warranted. Future studies could examine the current model on other vulnerable populations. Also, there was a lack of clinical cases for comparison. Future studies could also consider recruiting a clinical sample.

## Conclusions

Prior studies focused on the purposes of internet use that influence internet addiction as a whole concept, and not SNS addiction as a specific subtype of internet addiction. In this paper, the relationships between four categories of internet use purposes and SNS addiction were analyzed. The categories consisted of academic, economic, recreational, and social use. One of the major contributions of this study lies in exploring a significant negative influence of academic and informative purposes for using the internet on SNS addiction, in addition to addressing the high prevalence of this category of purposes in students. These findings have important implications for education and health providers, particularly in universities; we recommend that they try to improve students’ mental health and academic performance by providing opportunities for them to improve their IT literacy and skills. We can expand this study by recruiting varied students’ samples from other countries and cultures and conduct a comparative study among them in the future.

## Supplementary Information

**Additional file 1**. The survey.

## Data Availability

The data are available from the corresponding author on reasonable request.

## Abbreviations

IAT: Internet addiction test
SNS: Social networking site
IAT-SNS: Internet addiction test- social networking site version
SEM: Structural equation modeling
