# Supplementary material for: Purposes of internet use among Iranian university students: exploring its relationship with social networking site (SNS) addiction
Source: BMC Psychol. 2022 Mar 26;10:80. doi: 10.1186/s40359-022-00745-4 (PMC8966315; doi:10.1186/s40359-022-00745-4)
Supplement: Supplementary file 1 — Additional file 1. The survey. [file 40359_2022_745_MOESM1_ESM.docx]

**Additional file 1**

**The English and Persian versions of the survey questionnaire**

1. **Demographic information**

**1.1. English Version**

| 1. Age? …………. years. 2. Sex? Male 🞏 Female 🞏 3. Marital status? Single 🞏 Married 🞏 Other statuses 🞏 4. Part-time job? Yes 🞏 No 🞏 |
| --- |

**1.2. Persian Version**

| 1. سن: ........ سال 2. جنسيت: مرد 🞏 زن 🞏 3. وضعیت­ تاهل: مجرد 🞏 متاهل🞏 سایر🞏 4. آيا شغل پاره وقت داريد؟ بله خير  توضيحات:........................................... |
| --- |

1. **Purposes for internet usage measurement**
   1. **English Version**

How often do you use the Internet for …?

| **7**  **Extreme use** | **6** | **5** | **4** | **3** | **2** | **1** | **0**  **no use at all** |  |
| --- | --- | --- | --- | --- | --- | --- | --- | --- |
|  |  |  |  |  |  |  |  | 1. meeting new people |
|  |  |  |  |  |  |  |  | 1. following social and political news |
|  |  |  |  |  |  |  |  | 1. using SNSs |
|  |  |  |  |  |  |  |  | 1. online gaming |
|  |  |  |  |  |  |  |  | 1. online gambling |
|  |  |  |  |  |  |  |  | 1. relaxing |
|  |  |  |  |  |  |  |  | 1. listening to music or watching movies |
|  |  |  |  |  |  |  |  | 1. shopping |
|  |  |  |  |  |  |  |  | 1. making money |
|  |  |  |  |  |  |  |  | 1. using online transportation services |
|  |  |  |  |  |  |  |  | 1. learning |
|  |  |  |  |  |  |  |  | 1. information searching |
|  |  |  |  |  |  |  |  | 1. using email for educational needs |

- 1. **Persian Version**

**چه میزان از اینترنت برای موارد زیر استفاده میکنید؟**

|  | **صفر**  **(اصلا)** | **یک** | **دو** | **سه** | **چهار** | **پنج** | **شش** | **هفت**  **(خیلی زیاد)** |
| --- | --- | --- | --- | --- | --- | --- | --- | --- |
| **۱-ملاقات با افراد جدید** |  |  |  |  |  |  |  |  |
| **۲-دنبال کردن اخبار سیاسی و اجتماعی** |  |  |  |  |  |  |  |  |
| **۳-استفاده از شبکه های اجتماعی** |  |  |  |  |  |  |  |  |
| **۴-بازی آنلاین** |  |  |  |  |  |  |  |  |
| **۵-قماربازی آنلاین** |  |  |  |  |  |  |  |  |
| **۶-استراحت و کسب آرامش** |  |  |  |  |  |  |  |  |
| **۷-گوش دادن به موسیقی یا تماشای فیلم** |  |  |  |  |  |  |  |  |
| **۸-خرید آنلاین** |  |  |  |  |  |  |  |  |
| **۹-کسب درآمد** |  |  |  |  |  |  |  |  |
| **۱۰-حمل و نقل آنلاین** |  |  |  |  |  |  |  |  |
| **۱۱-یادگیری** |  |  |  |  |  |  |  |  |
| **۱۲-جستجوی اطلاعات** |  |  |  |  |  |  |  |  |
| **۱۳-استفاده از ایمیل برای مقاصد علمی** |  |  |  |  |  |  |  |  |

1. **Internet Addiction Test-Social Networking Sites version (IAT–SNS)**
   1. **English Version**

|  | How often… | Not Applicable | Rarely | Occasionally | Frequently | Often | Always |
| --- | --- | --- | --- | --- | --- | --- | --- |
| 1 | do you find that you stay on social networking sites longer than you intended? |  |  |  |  |  |  |
| 2 | do you neglect household chores to spend more time on social networking sites? |  |  |  |  |  |  |
| 3 | do you prefer the excitement of the social networking sites to intimacy with your partner? |  |  |  |  |  |  |
| 4 | do you form new relationships with follow social networking sites users? |  |  |  |  |  |  |
| 5 | do others in your life complain to you about the amount of time you spend on social networking sites? |  |  |  |  |  |  |
| 6 | do your grades or university work suffer because of the amount of time you spend on social networking sites? |  |  |  |  |  |  |
| 7 | do you check your social networking sites before something else that you need to do? |  |  |  |  |  |  |
| 8 | does your job performance or productivity suffer because of the social networking sites? |  |  |  |  |  |  |
| 9 | do you become defensive or secretive when anyone asks you what you do on social networking sites? |  |  |  |  |  |  |
| 10 | do you block out disturbing thoughts about your life with soothing thoughts of the social networking sites? |  |  |  |  |  |  |
| 11 | do you find that you find yourself anticipating when you will go social networking sites again? |  |  |  |  |  |  |
| 12 | do you fear that life without the social networking sites would be boring, empty or joyless? |  |  |  |  |  |  |
| 13 | do you snap, yell or act annoyed if someone bothers you while you are on social networking sites? |  |  |  |  |  |  |
| 14 | do you lose sleep due to late night log-ins on social networking sites? |  |  |  |  |  |  |
| 15 | do you feel preoccupied with the social networking sites when offline, or fantasize about being online? |  |  |  |  |  |  |
| 16 | do you find yourself saying “just a few more minutes” when be on social networking sites? |  |  |  |  |  |  |
| 17 | do you try to cut down the amount of time you spend on social networking sites? |  |  |  |  |  |  |
| 18 | do you try to hide how long you’ve been on social networking sites? |  |  |  |  |  |  |
| 19 | do you choose to spend more time on social networking sites over going out with others? |  |  |  |  |  |  |
| 20 | do you feel depressed, moody or nervous when you are offline, which goes away when you are back on social networking sites? |  |  |  |  |  |  |

- 1. **Persian Version**

| **همیشه** | **اغلب** | **به کرات** | **گاهی اوقات** | **بندرت** | **شامل حال من نمیشود** | **سوال** |  |
| --- | --- | --- | --- | --- | --- | --- | --- |
|  |  |  |  |  |  | **چند وقت یکبار برای زمانی طولانی­تر از آنچه که می­خواستی، در شبکه­های اجتماعی می­مانی؟** | **۱** |
|  |  |  |  |  |  | 1. **چند وقت یکبار از انجام کارهای خانه و ضروری صرف نظر می­کنی تا زمان بیشتری را در شبکه­های اجتماعی بمانی؟** | **۲** |
|  |  |  |  |  |  | 1. **چند وقت یکبار هیجان بودن در شبکه­های اجتماعی را به گذراندن وقت با دوست نزدیک (یا همسرت) ترجیح می­دهی؟** | **۳** |
|  |  |  |  |  |  | 1. **چند وقت یکبار به دنبال ایجاد ارتباط جدیدی با کاربران شبکه­های اجتماعی هستی؟** | **۴** |
|  |  |  |  |  |  | **چند وقت یکبار افراد خانواده یا دوستانت از مدت زمانی که در شبکه­های اجتماعی صرف می­کنی، ابراز ناراحتی می­کنند؟** | **۵** |
|  |  |  |  |  |  | **چند وقت یکبار مقدار زمانی که در شبکه­های اجتماعی صرف میکنی، موجب انجام ندادن وظایف تحصیلی یا درس خواندنت می­شود؟** | **۶** |
|  |  |  |  |  |  | **چند وقت یکبار قبل از انجام هر کار ضروری، شبکه­های اجتماعی خود را چک می­کنی؟** | **۷** |
|  |  |  |  |  |  | **چند وقت یکبار پرداختن به شبکه­های اجتماعی، موجب ایجاد مشکل در کارت می­شود؟** | **۸** |
|  |  |  |  |  |  | **چند وقت یکبار، وقتی اطرافیان از شما می­پرسند در شبکه­های اجتماعی چکار می­کنی، حالت دفاعی یا پنهان­کاری به خودت می­گیری؟** | **۹** |
|  |  |  |  |  |  | **چند وقت یکبار سعی می­کنی خودت را از دست افکار آزارنده در زندگی، با مشغول کردن خود در شبکه­های اجتماعی خلاص کنی؟** | **۱۰** |
|  |  |  |  |  |  | **چند وقت یکبار، دوباره وارد شبکه­های اجتماعی شدن را زودتر از زمانی که قصد داشتی، شروع می­کنی؟** | **۱۱** |
|  |  |  |  |  |  | **چند وقت یکبار نگران می­شوی که زندگیت بدون شبکه­های اجتماعی ملال آور، پوچ و بدون شادی خواهد بود؟** | **۱۲** |
|  |  |  |  |  |  | **هر چند وقت یکبار زمانی که در شبکه­های اجتماعی هستی و کسی مزاحمت می­شود، ناراحت می­شوی و ممکن است به طور ناگهانی فریاد بزنی؟** | **۱۳** |
|  |  |  |  |  |  | **چند وقت یکبار خوابت را به خاطر اینکه شب تا دیروقت در شبکه­های اجتماعی هستی، از دست می­دهی؟** | **۱۴** |
|  |  |  |  |  |  | **چند وقت یکبار وقتی که در در شبکه­های اجتماعی نیستی، احساس می­کنی که بدجوری فکرت در پی آن است و هوس رفتن شبکه­های اجتماعی را داری؟** | **۱۵** |
|  |  |  |  |  |  | **هر چند وقت یکبار به خودت می­گویی "فقط چند دقیقه دیگر" موقع رفتن به شبکه­های اجتماعی است؟** | **۱۶** |
|  |  |  |  |  |  | **هر چند وقت یکبار سعی می­کنی مدت زمانی را که در شبکه­های اجتماعی هستی را کوتاه کنی، اما نتوانستی؟** | **۱۷** |
|  |  |  |  |  |  | **هر چند وقت یکبار سعی میکنی مدت زمان در شبکه­های اجتماعی بودنت را از دیگران پنهان کنی؟** | **۱۸** |
|  |  |  |  |  |  | **هر چند وقت یکبار صرف کردن وقت بیشتر در شبکه­های اجتماعی را بر بیرون رفتن با دیگران ترجیح می­دهی؟** | **۱۹** |
|  |  |  |  |  |  | **هر چند وقت یکبار احساس می­کنی که وقتی در شبکه­های اجتماعی نیستی، افسرده یا عصبی هستی، در حالی­که این احساس­ها وقتی به شبکه­های اجتماعی می­روی از بین می­روند؟** | **۲۰** |
